# Financial Toxicity in Diabetes: The State of What We Know

**DOI:** 10.1007/s11892-025-01588-0

**Published:** 2025-05-13

**Authors:** Minal R. Patel

**Affiliations:** Department of Health Behavior & Health Equity, University of Michigan School of Public Health, 1415 Washington Heights, SPH 1, Room 3810, Ann Arbor, MI 48109-2029 USA

**Keywords:** Financial toxicity, Diabetes, Economic burden, Financial stress, Financial burden

## Abstract

**Purpose of Review:**

This systematic examination quantifies financial toxicity- the economic burden and related financial distress experienced by patients due to medical costs- in diabetes management globally, analyzing prevalence, mechanisms, and interventions across diverse healthcare systems and geographic contexts.

**Recent Findings:**

Data indicates 30–60% of diabetes patients experience financial toxicity, with household expenditures ranging from 5 to 40% of income on disease management, and demographic disparities evident. Current intervention strategies demonstrate limited efficacy, particularly in resource-constrained settings, while policy approaches show mixed results across economic contexts.

**Summary:**

Financial toxicity operates through four identified pathways: direct expenses, indirect costs (productivity/caregiver burden), insurance coverage limitations, and structural access barriers. Research priorities include developing validated measurement instruments for diabetes financial toxicity, implementing contextually appropriate interventions, and establishing causative relationships between financial burden and clinical outcomes through longitudinal studies.

## Introduction

Many terms are used in the literature to describe the epidemic of patient financial problems with healthcare, which is truly a global phenomenon with varying manifestations across healthcare systems worldwide. Financial toxicity—the economic burden and related financial distress experienced by patients due to medical costs^1^ —affects individuals with chronic conditions across diverse economies, healthcare systems, and social contexts [1–5]. While this phenomenon is known to be especially prevalent in market-based healthcare systems like the United States, financial toxicity represents a universal challenge that transcends national boundaries. With approximately 537 million adults living with diabetes worldwide—projected to rise to 783 million by 2045—understanding the financial burden associated with diabetes management has become increasingly important for population health globally [6].

In the United States, this financial toxicity grew over the 20th century with the rapid evolution of modern medicine and the nation’s complex healthcare financing system. In 2010, the launch of the Affordable Care Act was the first major national healthcare policy legislation since the passage of Medicare and Medicaid in 1965 that made a commitment to addressing affordability to meet medical care needs, though that commitment has not materialized to its full potential [7]. For instance, while one-third of U.S. adults are unable to afford a $400 medical expense without borrowing money^8^, similar financial vulnerability affects people with diabetes globally: in India, 71.5% of surveyed people with diabetes reported taking no diabetes medication due to affordability concerns; in Ghana, the National Health Insurance Scheme provides pre-mixed insulin but fails to cover glucose monitoring supplies; and even in high-income European countries with universal healthcare, indirect costs and gaps in coverage create financial strain, particularly for families managing Type 1 diabetes [8–11].

This review examines the current state of knowledge over the past five years regarding financial toxicity in diabetes care across diverse healthcare systems, economic contexts, and geographic regions, highlighting both common patterns and important differences in how financial toxicity manifests and is addressed worldwide.

## Conceptual Framework: Defining and Measuring Financial Toxicity

### Defining Financial Toxicity

Financial toxicity is a term that originated in the field of oncology and is considered a major side-effect of seeking cancer treatment—analogous to the toxicities of physical symptoms of treatment [1]. The term was first coined to bring attention to the often high out-of-pocket costs of cancer treatments and the corresponding adverse impacts on patients and their families [1].

Fenn et al. (2014) conceptualize financial toxicity as a composite measure that encompasses several dimensions of financial hardship, including out-of-pocket costs, productivity loss, asset depletion, medical debt, and bankruptcy [12]. The National Cancer Institute defines financial toxicity as the financial distress or hardship experienced by patients due to the cost of medical care [13]. This includes direct medical costs (e.g., treatment, medications) and indirect costs (e.g., lost income, travel expenses) which lead to financial strain and can adversely affect the patient’s quality of life and treatment adherence [13].

Lueckmann et al. (2021) expanded these definitions by highlighting that financial toxicity not only includes actual financial burden but also the anticipation and worry about potential financial distress [14]. This subjective financial distress can significantly impact the patient’s mental health and overall well-being, emphasizing the psychological dimension of financial burden that extends beyond objective economic measures [14].

The concept of financial toxicity has gained recognition as a critical concern across various chronic conditions, and notably diabetes [15–17]. Diabetes management requires ongoing medical monitoring, medication adherence, lifestyle modifications, and often advanced technologies—all carrying substantial costs. These expenses can create significant financial strain, leading to cost-related non-adherence (CRN) behaviors, worsened clinical outcomes, and diminished quality of life [18–22].

### Measuring Financial Toxicity in Diabetes

Financial toxicity in diabetes encompasses multiple dimensions: objective financial burden (direct and indirect costs), subjective financial distress (perceived hardship), and consequent behaviors including cost-coping strategies. Recent research has established metrics to quantify these dimensions, enabling more systematic analysis [23].

Patel et al. validated the COST-FACIT instrument for measuring financial toxicity, originally developed in cancer, in a clinical population with diabetes [23]. COST-FACIT demonstrated a two-factor structure with high internal consistency: general financial situation (7-items, α = 0.86) and impact of illness on financial situation (4-items, α = 0.73) [23]. In this study, the highest percentage of participants endorsed quite a bit/very much for “worry of financial problems in the future as a result of illness or treatment” (60%), and feeling that they “have no choice about money spent on care” (64%). Although nearly one-half endorsed quite a bit/very much for “I am able to meet my monthly expenses” (45%), just as many participants (42%) noted quite a bit/very much for “my out-of-pocket medical expenses are more than I thought they would be,” “I am financially stressed” (47%), and “I am frustrated that I cannot work or contribute as much as I usually do” (47%) [23]. Only 16% endorsed quite a bit/very much for “I am satisfied with my current financial situation [23].” These findings illustrate the multidimensional nature of financial distress that extends beyond simple ability to pay for care.

Patel et al. (2025) identified three distinct classes of financial toxicity (high, medium, and low) among people with diabetes, with a score of 26 on the COST-FACIT being the strongest threshold for distinguishing high versus medium/low financial toxicity [24]. This classification showed a positive predictive value of 76% and negative predictive value of 93%, providing a framework for identifying patients at greatest risk [24].

While validated instruments like COST-FACIT have been adapted for diabetes in Western contexts, different approaches to measuring financial burden have emerged in countries like India. Studies from India have focused on catastrophic health expenditure (CHE) thresholds, with research showing that 37.9% of Indian households with members who have diabetes experienced CHE at the 10% threshold, and nearly 10% fell below the poverty line due to diabetes-related out-of-pocket expenses [9].

### The Global Landscape of Financial Toxicity in Diabetes

Looking at financial toxicity in diabetes from a global perspective reveals several key patterns. Approximately 30–60% of people with diabetes worldwide experience some form of financial toxicity, though the severity and specific mechanisms vary significantly by healthcare system, income level, and geography [9, 24–28]. Across diverse countries, families affected by diabetes typically spend between 5 and 40% of household income on diabetes-related expenses, with the burden falling disproportionately on lower-income households who may spend as much as half their income on diabetes care in the most severe cases [26–28].

**Fig. 1:**
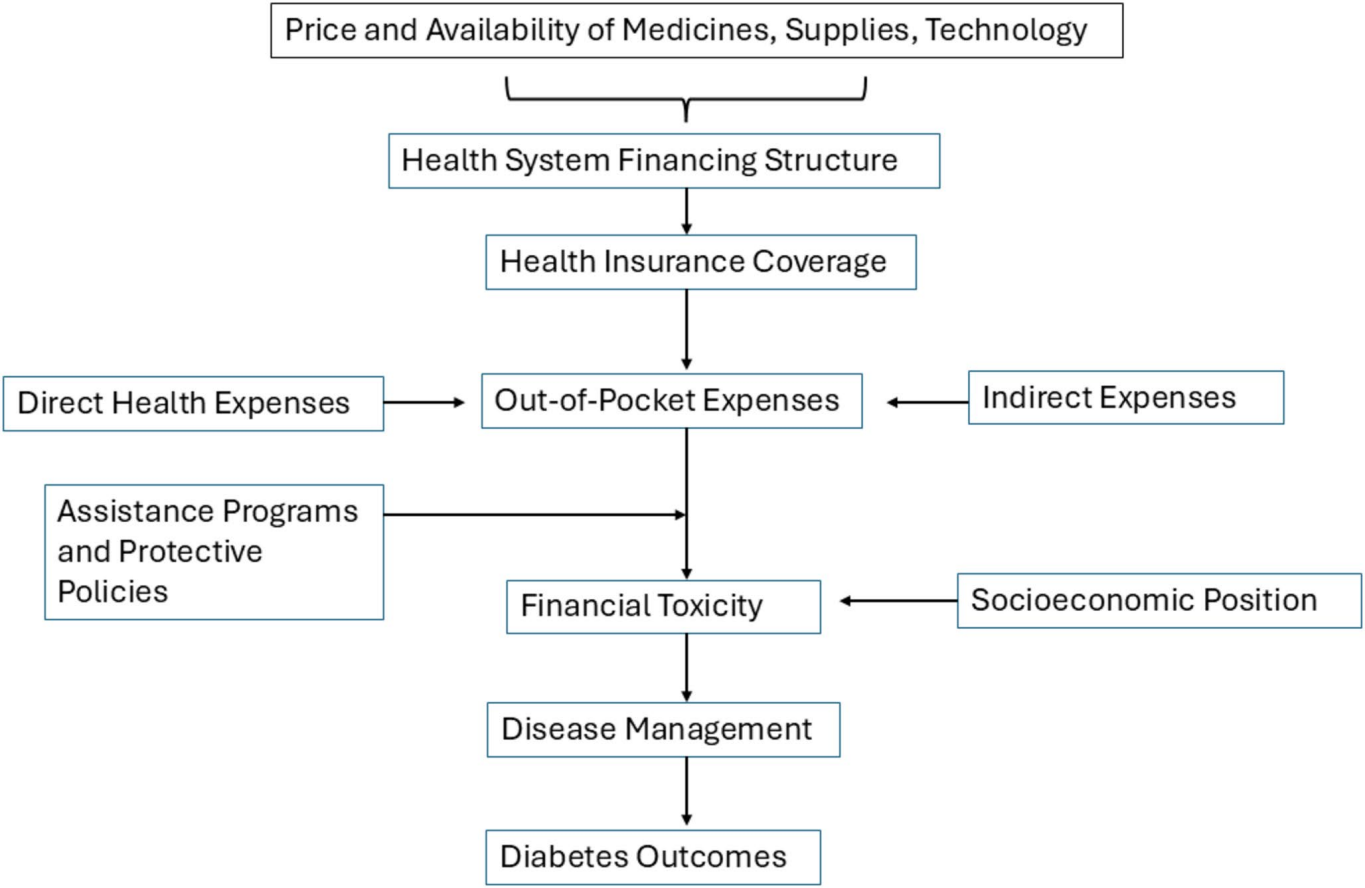
Conceptual Framework of Financial Toxicity in Diabetes

The primary drivers of financial toxicity operate through four key mechanisms that manifest differently across healthcare systems (Fig. 1):

1. **Direct out-of-pocket costs** for medications, supplies, and services create immediate financial pressure, affecting 40–70% of patients globally [29–31].
2. **Indirect costs** including transportation, lost productivity, and caregiver burden account for an often-overlooked 20–40% of total financial impact [11, 32–33].
3. **Coverage gaps** in both public and private insurance systems leave critical aspects of diabetes management unprotected—notably, diabetes technologies and outpatient services in many middle and low-income countries [10, 34–36].
4. **System-level structural barriers** including fragmented care delivery, complex eligibility requirements, and limited availability of affordable options compound these challenges [37–39].

Stark disparities characterize this landscape. Within countries, financial toxicity is consistently more severe among racial and ethnic minorities, women (particularly mothers of children with diabetes), rural residents, and those with multiple comorbidities [11, 40–41]. Between countries, the most extreme disparities are seen in years of life lost due to diabetes: approximately 22–23 years in high-income countries compared to 33–45 years in low-income settings [34]. Access to diabetes technology exemplifies these disparities, with adoption rates ranging from over 70% in some European countries to less than 3% in many African nations [34].

Meanwhile, the increasing cost trajectory of newer diabetes treatments and technologies threatens to widen existing gaps [22, 42–43]. This global landscape reveals financial toxicity not as an inevitable consequence of diabetes but as a systemic failure requiring coordinated policy, healthcare system, and individual-level interventions tailored to specific contexts [15, 44–45].

### Geographic Variations in Financial Toxicity

#### High-Income Countries

Even in high-income countries with robust healthcare systems, financial toxicity remains a significant challenge. Conway et al. (2024) examined disparities in diabetes technology uptake across countries and found that even among high-income countries, structural barriers such as stringent eligibility requirements by public and private insurers limited access to cost-saving technologies [34].

#### Low and Middle-Income Countries

Low and middle-income countries face unique challenges in addressing diabetes-related financial toxicity due to developing health insurance and coverage structures.

The financial burden of diabetes is especially pronounced in low-income countries where healthcare infrastructure may be limited and insurance coverage minimal [46–49]. Owusu et al. (2024) evaluated the effectiveness of Ghana’s National Health Insurance Scheme in providing financial protection to households with type 1 diabetes, concluding that the scheme steadily fails to meet the cost-reduction expectations of patients and their caregivers, particularly for non-insulin supplies like test strips and glucometers [10].

Conway et al. (2024) noted that in many low-income countries, particularly in Africa, diabetes technology market penetrance is negligible to nonexistent, with regions reporting among the highest years of life lost due to type 1 diabetes [30].

India presents a particularly stark case of financial toxicity in diabetes care despite recent government initiatives. The country’s healthcare financing structure exacerbates these challenges. While India has implemented large government-sponsored health insurance schemes like ‘Pradhan Mantri Jan Arogya Yojana (PMJAY),’ these primarily cover inpatient services and exclude outpatient care—a critical gap for diabetes management which relies heavily on ongoing outpatient services [9]. Additionally, poor availability of free or subsidized essential drugs in public health facilities compels individuals to purchase medicines from the open market, leading to higher out-of-pocket expenses or foregone treatments [9].

For advanced diabetes technologies like continuous glucose monitoring and insulin pumps, the situation is even more challenging. Only 10–15% of individuals with T1D in India use diabetes technology to manage glycemia [30].

### Mechanisms of Financial Toxicity in Diabetes

#### Direct Costs of Diabetes Management

**Fig. 2:**
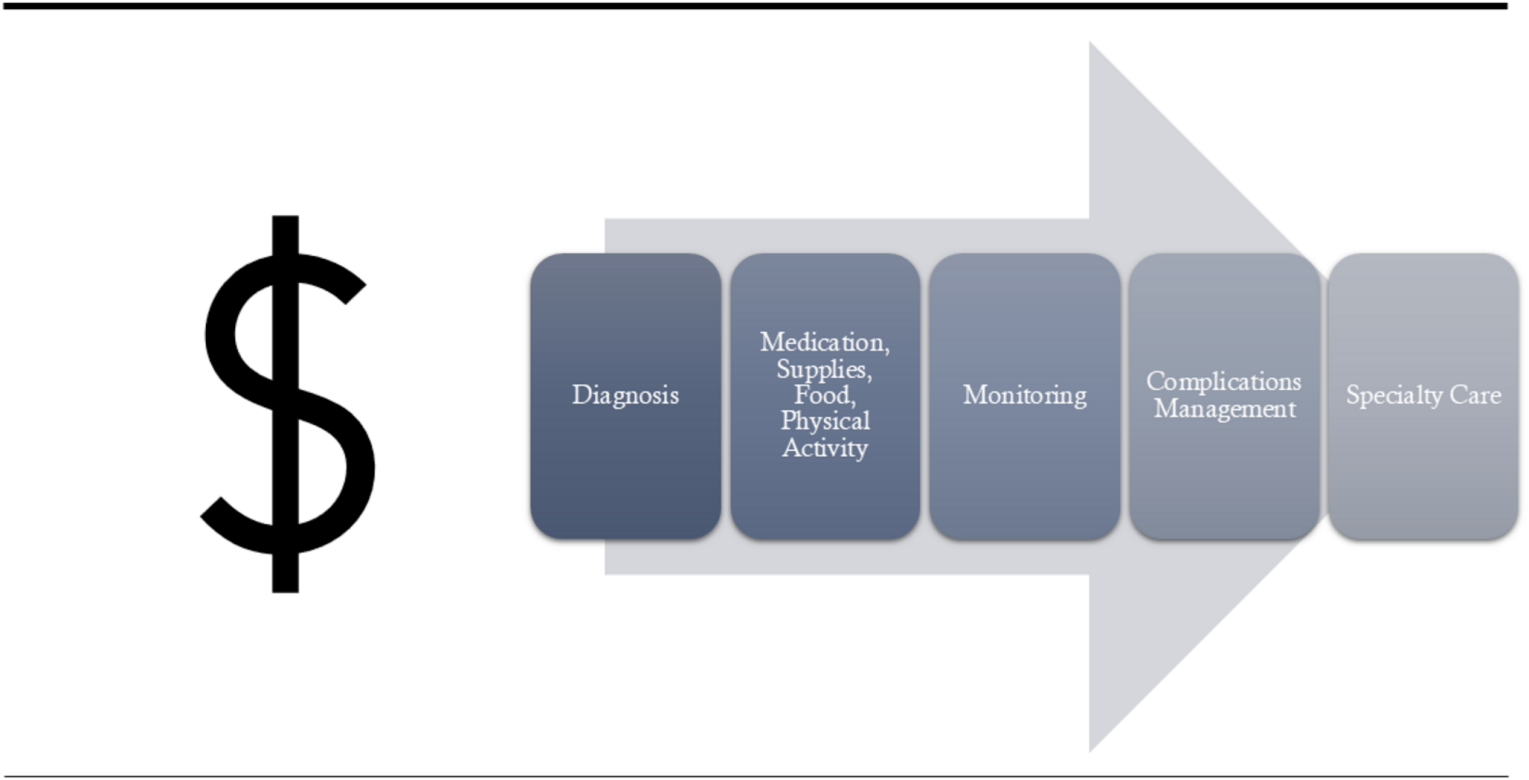
Financial Toxicity across the Diabetes Care Continuum

Direct costs for diabetes management constitute a substantial component of financial toxicity and include expenses for medications (particularly insulin), glucose monitoring supplies, devices, and healthcare services for routine disease management and treating complications (Fig. 2). These costs represent a cumulative burden. Recent literature reveals significant variations in these costs across different healthcare systems and populations.

#### Medication Costs

The cost and related insurance coverage of diabetes medications, particularly insulin, represents a major contributor to financial toxicity [50–53]. Julian et al. (2021) reported that out-of-pocket costs for insulin and diabetes-related supplies increased by 54% between 2005 and 2017 among privately insured patients in the United States, even after adjusting for inflation [29]. People with consumer-directed/high-deductible health plans experienced an even more dramatic 70% increase during this period [29].

Lin et al. (2023) found that while insulin usage remained stable over the past decade, total insulin expenditure almost doubled per person per year after the implementation of the Affordable Care Act in the US, regardless of insurance status [37]. For the uninsured, out-of-pocket insulin costs increased from $1,678 per person per year to $2,800 per person per year [37].

Non-insulin medications also contribute significantly to financial burden with diabetes. Quach et al. (2024) highlighted that median annualized out-of-pocket spending for SGLT2 inhibitors and GLP-1 agonists was higher than for insulin or DPP-4 inhibitors among commercially insured individuals in the US [42]. People with diabetes taking three or more classes of diabetes medications faced particularly high costs, with the top decile spending nearly $3,000 annually out-of-pocket for medications alone [42].

In middle and low-income countries, medication costs represent an even greater relative burden [49, 54–55]. Mohammed et al. (2024) found that in Ethiopia, none of the lowest-priced generic diabetes medicines were affordable in either public or private healthcare settings, with prices in private pharmacies significantly exceeding reference prices [30].

While insulin costs have risen dramatically in the United States, medication affordability represents an even greater relative burden in India. Rawat et al. (2024) found that families in Delhi with a family member managing diabetes may lose 10–40% of their gross family income to diabetes care expenses [26]. Moreover, a recent study examining affordability of essential medicines found that 51.4% of Indian households with diabetes members were unable to afford insulin and 24.6% couldn’t afford metformin, with the highest unaffordability reported in rural areas and among the lowest income tertile [9]. Perhaps most alarmingly, among 2,972 surveyed diabetes patients in India, 71.5% reported taking no diabetes medication at all due to cost barriers [9].

### Monitoring Supplies and Devices

Beyond medications, costs for glucose monitoring supplies and devices constitute a significant portion of direct expenses [22]. Fauzi et al. (2024) detailed the costs associated with self-monitoring blood glucose (SMBG) devices in Indonesia, noting that glucometers cost between 19.21 and 38.40 USD, with a pack of 25 glucose strips ranging from 3.84 to 7.68 USD [35]. With a minimum requirement of four glucose tests per day, this totals approximately 38.40 USD per month—a substantial burden in a country where the monthly average household income was 203.52 USD in 2023 [35].

Lin et al. (2023) found that in China, approximately 89.5% of older adults with diabetes were reluctant to have their blood glucose monitored for economic reasons [56]. Blood glucose test strips were not covered by medical insurance, requiring patients to bear these costs themselves [56].

### Healthcare Services

Costs for healthcare services—including consultations, laboratory tests, and hospitalizations—also contribute significantly to financial toxicity among people with diabetes worldwide [57]. Rawat et al. (2024) examined out-of-pocket direct costs of ambulatory care for type 2 diabetes in Delhi, India, finding that families who have a member managing diabetes may lose 10–40% of their gross family income to diabetes care expenses [26].

For people with complications, these costs escalate dramatically [58–62]. Seshadri et al. (2024) reported that among patients with diabetic foot ulcers in South India, the median total annual out-of-pocket expenditure was ₹29,775 ($378.14), with 68.8% of participants facing catastrophic expenditure (> 10% of annual income) for management of these complications [33]. India has been described as ‘the most expensive country for diabetic foot ulcer care,’ with an estimated 5.7 years (68.8 months) of an average patient’s income required to pay for complete DFU therapy [33, 61].

Phrommintikul et al. (2022) found in Thailand that the average total out-of-pocket outpatient cost for diabetes was THB 22,874 ± 38,066 ($759 ± 1,264) for the first year, while out-of-pocket inpatient expenditure averaged THB 160,790 ± 411,607 ($5,338 ± 13,666), with complications dramatically increasing costs [57].

### Indirect Costs and Broader Economic Impact

Beyond direct healthcare expenses, indirect costs significantly contribute to the overall financial toxicity of people managing diabetes [15]. These indirect expenses include productivity losses due to missed work or reduced capacity, substantial caregiver burden affecting family finances, and transportation expenses to healthcare facilities. These factors often receive less attention in policy discussions but can substantially impact household finances [32].

### Productivity and Employment Impacts

Diabetes-related financial toxicity significantly affects employment and productivity [11]. Dehn-Hindenberg et al. (2021) documented substantial occupational consequences for families of children with type 1 diabetes in Germany, with 15.1% of mothers stopping work entirely and 11.5% reducing working hours following their child’s diagnosis [11]. While mothers’ occupational status was significantly reduced, fathers’ working status hardly changed, highlighting gender disparities in caregiver burden. Nearly half of these families (46.4%) reported moderate to severe financial losses [11].

### Transportation and Time Costs

Transportation costs represent a significant but often overlooked component of diabetes-related financial burden [32]. They represent both location of healthcare facilities in general, and those that provide covered services and supplies based on insurance status.

Faizi et al. (2025) found that the majority of people with diabetes in Indonesia living in rural areas had to pay travel expenses related to diabetes care, reflecting the geographic disparities in access to specialized care [28]. Oyando et al. (2023) noted that in Kenya, transport costs represented 23% of direct costs to access diabetes care [32]. These costs were particularly burdensome for rural residents who often needed to travel to urban centers for specialized care.

Time costs—including time spent traveling to appointments, waiting for care, and managing diabetes at home—also contribute to the overall burden [11]. While difficult to quantify monetarily, time costs represent real opportunity costs for patients and families, particularly in contexts where hourly wages or daily income is lost due to care-seeking behavior [28].

### Caregiver Burden

Caregiver burden represents another significant indirect cost, particularly for children, elderly patients, and those with complications. Marwi et al. (2024) studied the social and financial burden on families of pediatric patients with type 1 diabetes in Saudi Arabia, highlighting multifaceted caregiver burden dimensions, including financial, physical, social, spiritual, and emotional stresses [63]. The study identified financial difficulties such as stopping savings, taking loans, and not buying non-essential commodities, with a strong positive correlation between stopping leisure activities and financial stress related to caregiving [63]. This burden affects not only household finances but also caregivers’ mental and physical health [11].

The financial implications of caregiver burden vary significantly across healthcare systems. Research from India reveals particularly severe consequences. Rohilla et al. (2022) found that among North Indian families of children with T1D, 30.3% spent more than 50% of their total family income on diabetes care, 36.6% had to borrow money, and for 8.2% of families, out-of-pocket expenditures exceeded income from all sources, pushing them toward financial collapse [27]. The authors characterized the situation as ‘almost on the verge of losing sustainability.’

### Interventions to Address Financial Toxicity

Interventions to address financial toxicity operate at multiple levels within healthcare ecosystems. Policy-level approaches typically target medication pricing and insurance coverage, while healthcare system interventions focus on redesigning care delivery models and providing patient navigation support. At the individual level, interventions aim to equip patients with tools and resources to better manage the financial aspects of their condition.

### Policy-Level Approaches

Policy interventions, particularly those addressing medication costs, have been implemented across various jurisdictions. Twenty-five states in the US have implemented insulin out-of-pocket cost caps, and the Inflation Reduction Act imposed a $35/month cap on insulin for Medicare beneficiaries [64]. Studies show state-level caps generally reduce out-of-pocket spending but have mixed effects on insulin use.

Garabedian et al. (2024) evaluated the effect of state insulin out-of-pocket caps on insulin use and costs among commercially insured persons with diabetes [65]. While these caps were associated with reduced insulin out-of-pocket costs (particularly for those with health savings accounts), they did not lead to overall increases in insulin use, suggesting additional barriers beyond direct cost influence insulin utilization.

Giannouchos et al. (2024) found more nuanced effects in Colorado, where legislation capping out-of-pocket costs at $100 per prescription for a 30-day supply of insulin was associated with increased adherence to insulin therapy, but only among users who spent $100 or more out-of-pocket at least once in the pre-policy period [66]. This suggests that cost caps may be most effective for those facing the highest financial barriers.

Anderson et al. (2024) similarly found no significant increase in quarterly insulin claims following state-level caps on insulin out-of-pocket spending among commercially insured enrollees [67]. The authors suggested that state-level caps on insulin out-of-pocket spending did not significantly increase insulin use, potentially because out-of-pocket expenses were already lower than cap amounts for many patients.

Policy interventions to address financial toxicity extend beyond high-income countries. In India, which bears the world’s largest diabetes burden, the government has introduced the Pradhan Mantri Bhartiya Janaushadhi Pariyojana to provide access to quality generic medicines at affordable prices [9]. This initiative aims to significantly reduce the financial burden of diabetes medications by making lower-cost alternatives more widely available. The impact of such programs is potentially substantial—adopting generic medicines could significantly reduce the financial burden caused by diabetes management [9].

Additionally, small-scale studies have demonstrated the potential of technology access initiatives in India: when continuous glucose monitoring was provided to children with T1D in resource-poor areas, HbA1c was significantly reduced from 11.23 to 10.14%, suggesting that addressing financial barriers to technology access could yield important clinical benefits [34].

### Healthcare System Approaches

Healthcare system interventions aim to address financial toxicity through changes in care delivery and support structures. Recent evidence suggests these approaches face significant implementation challenges.

McQueen et al. (2024) conducted a randomized controlled trial testing the effects of a social needs navigation intervention on health outcomes among Medicaid members with type 2 diabetes [68]. While they found no group differences in HbA1c tests and values or other outcomes, the study highlighted the persistence of social needs despite intervention efforts, suggesting that navigation alone may be insufficient to address underlying structural barriers.

Similarly, Patel et al. (2024) evaluated the effectiveness of the CareAvenue intervention for unmet social needs among adults with diabetes, finding no significant improvements in HbA1c lowering, met needs, cost-related non-adherence, or perceived financial burden compared to control participants [69]. These findings suggest that more comprehensive approaches addressing both healthcare delivery and broader social determinants may be necessary to meaningfully reduce financial toxicity.

### Individual-Level Approaches

At the individual level, various interventions aim to help patients navigate financial challenges. Herrick et al. (2021) found that cost conversations and cost coping strategies were common among individuals with diabetes [45]. The study highlighted that individuals with diabetes were more likely to report spending less on basic needs to pay for medications, with many reducing food expenditures—a trade-off potentially counterproductive for diabetes management.

Herges et al. (2021) provided guidance for patients and primary care clinicians on easing the financial burden of diabetes management, emphasizing the importance of clinician awareness of financial impact and strategies to reduce costs, including medication selection, assistance programs, and insurance optimization [22]. Their framework emphasized proactive cost discussions between clinicians and patients, suggesting that increased awareness of financial burden among healthcare providers could lead to more cost-conscious prescribing and treatment recommendations.

### Research Agenda: Addressing Knowledge Gaps

Despite growing literature on financial toxicity in diabetes, significant knowledge gaps remain. Priority research areas include:

1. Developing standardized measurements of diabetes-specific financial toxicity that can be implemented across diverse settings.
2. Conducting longitudinal studies examining the long-term impacts of financial toxicity on clinical outcomes, quality of life, and economic well-being.
3. Conducting rigorous evaluations of policy, system, and individual-level interventions, particularly in diverse populations and settings. For example, policies that accelerate the availability of generic medicines, ensure supply of essential diabetes medicines and diabetes-related technologies and devices should be explored further.
4. Assessing how emerging diabetes technologies affect financial toxicity across different populations and payment systems.
5. Expanding research on how financial toxicity affects family systems, including caregivers of people with diabetes.
6. Identify effective strategies for implementing and scaling interventions to reduce financial toxicity in different contexts.

## Conclusion

Financial toxicity represents a significant challenge in diabetes care globally, affecting patients across diverse healthcare systems and socioeconomic contexts. This review has synthesized recent literature to illustrate the multidimensional nature of financial toxicity, its varying manifestations across populations and geographies, and emerging approaches to address this critical issue.

The literature clearly shows that diabetes imposes a substantial financial burden on individuals and families, with serious consequences for medication adherence, health outcomes, and quality of life. The financial impact extends beyond direct medical costs to include significant life disruptions and difficult trade-offs between medical care and basic necessities.

Key findings from this review indicate that financial toxicity in diabetes is widespread, with high prevalence of out-of-pocket spending, cost-related non-adherence, and financial stress across countries. Both direct costs—particularly for medications, monitoring supplies, and healthcare services—and indirect costs—including productivity losses, transportation expenses, and caregiver burden—constitute major contributors to overall financial burden, though their relative importance varies by context. Our analysis reveals significant disparities in financial toxicity across geographic regions, healthcare systems, and population subgroups, highlighting the need for contextually tailored approaches.

Future efforts should focus on developing standardized measures of financial toxicity, implementing and evaluating contextually appropriate interventions, and ensuring that solutions address not only direct costs but also the broader financial impacts of living with diabetes. With coordinated action across sectors, the significant burden of financial toxicity in diabetes can be meaningfully reduced.

## Key References

**Nanda M, Sharma R. Financial burden of seeking diabetes mellitus care in India: Evidence from a Nationally Representative Sample Survey. Health Care Sci. 2023 Oct 4;2(5):291–305. This study reveals that among 2,972 surveyed diabetes patients in India, 71.5% reported taking no diabetes medication at all due to cost barriers.
**Patel MR, Zhang G, Heisler M, Song PXK, Piette JD, Shi X, Choe HM, Smith A, Resnicow K. Measurement and Validation of the Comprehensive Score for Financial Toxicity (COST) in a Population With Diabetes. Diabetes Care. 2022 Nov 1;45(11):2535–2543. This research validated a reliable tool to measure financial toxicity in diabetes patients.
*Fauzi M, Fadiana G, Nadira D, Angela A, Puteri HA, Pulungan A. Pediatric Type 1 Diabetes Care in Indonesia: A Review of Current Challenges and Practice. J Clin Res Pediatr Endocrinol. 2024 Nov 27. doi: 10.4274/jcrpe.galenos.2024.2024-9-4. This review identifies significant gaps in Indonesia’s diabetes care system where essential monitoring devices like glucometers and glucose strips are not covered by national health insurance, creating substantial financial burdens for families.
**Conway RB, Snell-Bergeon J, Honda-Kohmo K, Peddi AK, Isa SB, Sulong S, Sibomana L, Gerard Gonzalez A, Song J, Lomax KE, Lo CN, Kim W, Haynes A, de Bock M, Burckhardt MA, Schwab S, Hong K. Disparities in Diabetes Technology Uptake in Youth and Young Adults With Type 1 Diabetes: A Global Perspective. J Endocr Soc. 2024 Nov 28;9(1):bvae210. doi: 10.1210/jendso/bvae210. This global survey reveals that economic barriers are the primary obstacle to diabetes technology access worldwide, with striking disparities between high-income and lower-income countries in the adoption of continuous glucose monitoring and insulin delivery systems.
*Quach J, Midlam C, Sell C, Levin-Scherz J. Out-of-pocket costs for diabetes medications in employer-sponsored health insurance plans. Am J Manag Care. 2024 Mar;30(3):107–108. doi: 10.37765/ajmc.2024.89510. This analysis demonstrates that out-of-pocket costs for non-insulin diabetes medications can be extremely high for patients with employer-sponsored insurance, with those taking multiple drug classes facing potential annual costs approaching $3,000.
*Garabedian LF, Zhang F, Costa R, Argetsinger S, Ross-Degnan D, Wharam JF. Association of State Insulin Out-of-Pocket Caps With Insulin Cost-Sharing and Use Among Commercially Insured Patients With Diabetes: A Pre-Post Study With a Control Group. Ann Intern Med. 2024 Apr;177(4):439–448. doi: 10.7326/M23-1965. This study found that state-level insulin cost caps reduced out-of-pocket expenses by 17.4% overall (with greater reductions of 40% for those with more generous caps), but did not significantly increase insulin utilization.

## Data Availability

No datasets were generated or analysed during the current study.
